# Measuring Early Cortical Visual Processing in the Clinic

**DOI:** 10.1177/2041669517702915

**Published:** 2017-05-17

**Authors:** Linda Bowns, William H.A. Beaudot

**Affiliations:** University of Cambridge, Cambridge, UK; KyberVision Japan LLC, Sendai, Japan

**Keywords:** Component Extraction and Motion Integration Test, clinical test, visual cortex, motion integration, component extraction

## Abstract

We describe a mobile app that measures early cortical visual processing suitable for use in clinics. The app is called *Component Extraction and Motion Integration Test* (CEMIT). Observers are asked to respond to the direction of translating plaids that move in one of two very different directions. The plaids have been selected so that the plaid components move in one of the directions and the plaid pattern moves in the other direction. In addition to correctly responding to the pattern motion, observers demonstrate their ability to correctly extract the movement (and therefore the orientation) of the underlying components at specific spatial frequencies. We wanted to test CEMIT by seeing if we could replicate the *broader tuning* observed at low spatial frequencies for this type of plaid. Results from CEMIT were robust and successfully replicated this result for 50 typical observers. We envisage that it will be of use to researchers and clinicians by allowing them to investigate specific deficits at this fundamental level of cortical visual processing. CEMIT may also be used for screening purposes where visual information plays an important role, for example, air traffic controllers.

## Introduction

Two-dimensional images can be uniquely described in terms of a collection of sinusoidal luminance patterns that vary in orientation, spatial frequency, phase, and contrast. These sinusoidal patterns (components) may be thought of as an *alphabet* of the visual image, and extracting these patterns from the image is critical to early visual processing. The visual systems of humans and other mammals have evolved to extract these patterns at a local level to efficiently encode the vast number of visual images perceived (Campbell & Robson, 1968). There have been a number of methods developed for measuring early visual processing performance in humans (Beaudot & Mullen, 2006; Blakemore & Nachmias, 1971; Movshon and Blakemore, 1973; Ringach, 1998). These methods, however, are aimed at understanding the underlying physiological mechanisms and can involve complex stimuli, lengthy observations, and training the observers.

Currently, there are no simple psychophysical tests to measure early visual cortical performance that would be quick, simple, and robust. Brain imaging is expensive and would not provide the specific detailed information that one would get from a traditional psychophysical approach, where an observer has to prove that they can respond accurately to a stimulus. A forced-choice task is often used in psychophysics, where some property of the stimulus is varied and makes it more or less difficult to respond to. The percentage of correct responses follow a psychometric function where the threshold is determined at which observers no longer respond at chance level. By convention, this is the 75% point on the function, and this is the detection threshold that is frequently provided as proof that an observer has accurately detected the stimulus during a forced-choice task. This traditional approach has not been designed for clinical use, so we have chosen to develop a completely new method using some of this carefully developed knowledge from vision research.

## Materials and Methods: Critical Properties of CEMIT Stimuli

**Figure 1.**
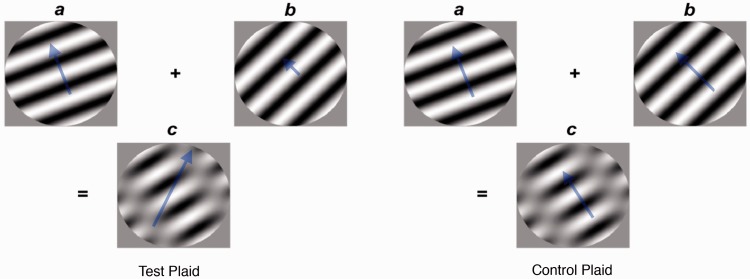
Illustration of the component and pattern velocities for the test and control patterns; (a) and (b) illustrate the two components used to create the plaid shown in (c). The arrows indicate their veridical velocities (enlarged for clarity). The plaid velocity in (c) is computed using the Intersection of Constraints rule. Mirror images of the test and control plaids are used to counterbalance with respect to direction.

When two moving sinusoidal components are combined to form a plaid pattern, perceived pattern direction is different from either of its two component directions. Pattern direction is however predictable from the properties of individual components (Adelson & Movshon, 1982; Bowns, 1996; Bowns & Alais, 2006; Yo & Wilson, 1992). Figure 1 illustrates two examples of the relationship between component velocity and pattern velocity. Images of two components are shown Figure 1(a) and (b); an arrow illustrates their veridical velocity, direction (angle of the arrow), and speed (length of the arrow). Their combined pattern is shown in Figure 1(c) (the plaid), together with the resulting pattern direction as perceived by a typical observer using a combination method known as *the intersection of constraints rule* (Adelson & Movshon, 1982). The difference between the component direction and pattern direction is unusually large, therefore making it easily distinguishable and an optimal plaid in a forced-choice task. The task is to respond to the motion direction as moving in a clockwise or anticlockwise direction relative to a reference line, equidistantly positioned between the component and pattern directions. Although it has been shown that pattern direction can change with the duration of the stimulus (Yo & Wilson, 1992), the specific plaids illustrated in Figure 1 remain stable at different durations (Bowns, 1996; Bowns & Alais, 2006). Our test plaids have component orientations that vary by just 23°. This is an important property because this difference is optimal in revealing broader tuning of orientation at different spatial frequencies. It is known that observers fail to respond to the plaid motion at low spatial frequencies and instead perceive component motion suggesting broader tuning to orientation, that is, an inability to perceive two separate components (Bowns & Beckett, 2010).

## Method

We wanted to test CEMIT by seeing if we could replicate broader tuning of orientation filters at low spatial frequencies. This has been estimated to be at spatial frequency values less than 0.5 cpd (Bowns and Beckett, 2010). Therefore, the range of spatial frequencies used was a four-octave range from 0.2 cpd to 1.6 cpd. We used the vertical orientation of the plaids as shown in Figure 1. We refer to this as the *standard test* because this is the condition we believe would be most suitable for use in clinics.

All observations were carried out under a variety of environmental conditions in order to simulate clinical rather than laboratory conditions. Observers viewed the display with two eyes. In a clinical context, because the test is easy and fast, it could be repeated monocularly if the subject shows abnormal performance in the binocular condition. Distance was always measured using the front-facing camera of the iPad (see Appendix for method and accuracy of calibration) and observers were asked to maintain their distance to the screen, to fixate the red central dot during each trial, and to only respond to motion. The accuracy of the distance was measured independently during testing and found to be very accurate, as described in the Appendix, that is, within 1 cm at a distance of up to 70 cm. Observers had normal or corrected vision and were randomly selected from a student population. Observers were asked if they had normal or corrected vision as determined by their own optician. However, as the spatial frequency range was relatively low and the orientation difference of the components greater than 20° across the range tested, it would be highly unlikely that our results would be influenced by minor eye problems such as astigmatism or mildly blurred vision. (CEMIT contains a form for entering clinical and other individual information.) The CEMIT mobile app was run on the iPad, Model MC705B, running iOS 5.1.1. Viewing distance was determined by CEMIT using the front facing camera, and the display used CEMIT’s Gamma correction (see Appendix).

### Stimuli

There were four types of plaids: one test plaid, one control plaid, and the mirror image plaids of these two. Each of these four plaids was presented at a range of different spatial frequencies. The mirror image plaids controlled for direction bias. The control plaid ensured that the spatial properties remained similar to the test plaid while the motion direction was in a similar direction to the component directions. This is a *Type 1 plaid* (Ferrera & Wilson, 1991) where the pattern direction falls between the components and in this case was equal to the vector average of the components. If observers responded to the spatial properties, results for the control plaid would be the same as the test plaid and therefore incorrect. A comparison of pattern velocities and spatial properties of the test and control plaids is shown in Figure 1. For the test plaid, the vector average of the component directions and the pattern direction had equal clockwise or anticlockwise direction relative to a comparison line. The test plaid pattern that had the clockwise pattern direction comprised components with orientation 202° and 225° where 0° was set at the horizontal. The mirror test plaid was constructed using orientation values −202° and −225° and had an anticlockwise pattern direction. The first frame of each component was in cosine phase and was shifted through a fixed phase shift on subsequent frames to create the motion. The phase shifts used were 40° and 18° respectively, thus creating a *Type II plaid* (Yo & Wilson, 1992), where the pattern direction is known to be perceived in a direction predicted by the *intersection of constraints* direction of 61.7° at short and long durations (Bowns, 1996; Bowns and Alais, 2006), and the vector average of the component directions is 119.1° Therefore, there was a large difference between the pattern and component directions of 57.4°, making the task easier than other plaid stimuli, and in addition keeping variables that affect motion direction constant. For a discussion of these variables and the equations used to generate the stimuli, see Bowns and Beckett (2010). The plaids moved in a circular aperture, with a viewing angle of 8° Each of the four plaids was presented at four different spatial frequencies spanning a four-octave range from 0.2 cpd to 1.6 cpd. Speed remained constant at 2°/s. Each moving plaid was presented 10 times. Therefore, an observer was presented with 160 trials for each test, which took approximately 3 to 4 minutes. Each moving pattern appears for 0.5 seconds. All experiments were carried out in accordance with The University of Nottingham ethics and risk assessment procedures, “in accordance with the Code of Ethics of the World Medical Association (Declaration of Helsinki).” Consent was obtained for experimentation with human subjects.

### Procedure

Once the observer’s distance had been computed by CEMIT, the observer was asked to press a green arrow to begin the test. A red fixation dot appeared along with a green dot that indicated the virtual reference line. The plaids were presented pseudo randomly. Observers used the touch screen to indicate the direction of the movement relative to the virtual line. Their response triggered the next trial.

## Results

**Figure 2.**
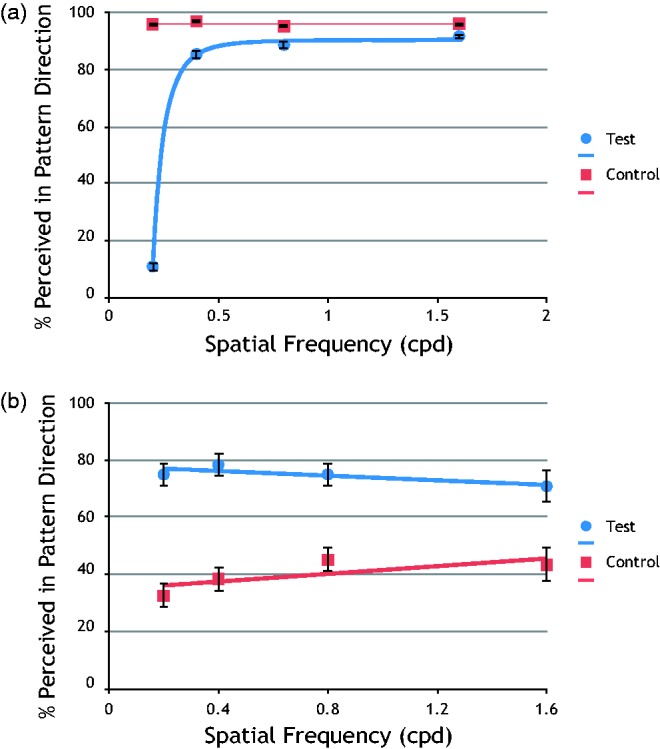
(a) CEMIT results for 50 typical observers. The percentage of responses perceived in the pattern direction (IOC) is plotted against spatial frequency. The dramatic shift at low spatial frequencies is as predicted. Results for the control are close to 100% correct and show that observers were not responding to spatial information. (b) Results for six observers unable to do the task. Although some of their test results are slightly above chance at lower spatial frequencies, their control results suggest that they were using spatial information more often at these frequencies.

Figure 2(a) shows the average results for 50 observers for the vertical orientation test, that is, the version we refer to as the *standard test*. The percent perceived in the pattern direction (as determined by the Intersection of Constraints) was plotted against the spatial frequency. As there was no precedent for a theoretical curve fitting to this type of data, we have used a power function to fit the data; error bars show the standard error of the mean. The average results show that results are similar to those previously reported and were robust. There is a dramatic change in response where observers failed to resolve the orientation of the components below 0.5 cpd as predicted. In addition to the test data, we show the control data. Response to the control plaids is close to 100% in the pattern direction and therefore shows that observers were not using spatial information rather than motion. It is known that the ratio of the spatial frequency to the stimulus envelope can affect the spatial frequency bandwidth; however, when this ratio is held constant, the broader tuning to orientation remains and therefore cannot explain our results (Bowns & Beckett, 2010). Control data also show that the observers responded appropriately to the spatial frequencies and other properties of the components shared with the test stimuli. Data from the mirror plaids were combined with the test and control plaids. Observers who failed to obtain greater than 75% on the control have been removed from the data because they either cannot do the task or they are using spatial rather than movement information. The results for these six observers are shown in Figure 2(b) and are mostly around chance performance. Although their test results are sometimes above chance at the lower spatial frequencies, they could achieve this by using spatial information. This relationship can be seen in the graph, when performance is low on the control, indicating the use of spatial information, it is higher on the test.

**Figure 3.**
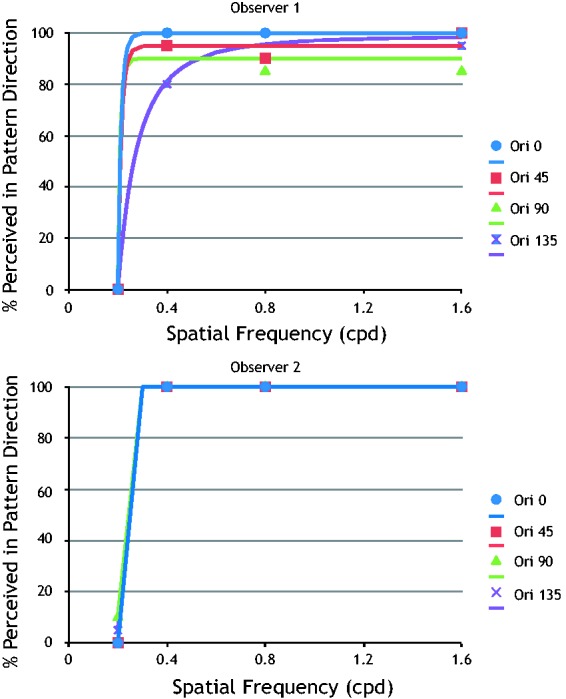
CEMIT results for two individual observers at four different plaid orientations. Both observers show the dramatic shift at low spatial frequencies at all four plaid orientations.

To see if our results would differ as a function of plaid orientation, we repeated our test at four different orientations for two observers. Observer 1 was a naive observer and had never performed any psychophysical or similar task before. Observer 2 was a trained observer. Results for the two observers are shown in Figure 3(a) and (b), respectively. The characteristic dramatic change in performance at low spatial frequencies is clear for both individual observers at all four plaid orientations.

## Discussion

### Advantages of Our Approach

#### Suitable for mobile clinical use

We are able to measure performance for a plaid series covering a four-octave range of spatial frequencies in approximately 3 to 4 minutes, and the task is easy. CEMIT was implemented as a comprehensive mobile app for iOS devices (iPhone, iPod touch, and iPad), with the abilities to specify and preview the stimuli properties, design and run the test, and save and export the experimental results. It provides a method for computing the distance between the observer and the screen using the front-facing camera and has a facility for gamma correction.

#### Excellent control of stimulus parameters

Our results cannot be attributed to first order, second order, vector averaging, intersection of constraints, or contrast; these are held constant as a function of spatial frequency. Spatial properties and direction bias are specifically controlled for.

#### Orientation tuning of filters at varying spatial frequencies

Broad tuning of orientation leads to loss of information. This can be measured using CEMIT. Results for typical observers described above show robust data on the limitations of orientation tuning at low spatial frequencies. CEMIT may reveal different tuning patterns for specific clinical groups.

#### Pattern motion integration

Results from CEMIT provide a clear indication of an observers’ ability to integrate component motion into pattern motion.

### General Discussion

Testing using CEMIT was fast, for some observers it took just 3 minutes. CEMIT has the potential to provide very specific information at the cortical level and also provide valuable information about a specific deficit. Results from CEMIT reveal information about the limits of performance that we could use as performance markers to explore individual differences across populations. We hope to use these limits, together with specific deficits to investigate both typical and clinical populations. Indeed various visual impairments due to neural dysfunctions in several brain disorders or diseases can be investigated psychophysically (Beaudot, 2009). The aim of our test is to seek individual differences, so we were pleased that around 10% of our observers performed differently from the typical observers. We do not set out in this paper to address specific clinical groups and therefore cannot identify why they performed atypically.

There are a number of clinical conditions where visual problems have been identified. For example, poor readers or people who have some types of dyslexia have problems detecting motion, but the precise nature of the motion problem has yet to be identified, that is, whether or not it occurs at specific directions or spatial frequencies, or varies with observers (Cornelissen, Richardson, Mason, Fowler, & Stein, 1995; Demb, Boynton, Best, & Heeger, 1998; Everatt, Bradshaw, & Hibbard, 1999; Ridder, Borsting, & Banton, 2001). Visual processing problems also occur in observers with autism spectrum disorder (ASD), but again little is known about the precise nature of the problem (Bertone, Mottron, Jelenic, & Faubert, 2003; Bertone & Faubert, 2006; Kaiser & Shiffrar, 2009; Koldewyn, Whitney, & Rivera, 2011; Milne et al., 2002). Similarly, problems with early cortical visual processing have also been identified in patients with Alzheimer’s disease (Leuba & Kraftsik, 1994) and Parkinson’s disease (Trick, Kaskie, & Steinman, 1994). CEMIT may help to provide a more precise description of any deficits of early cortical visual processing in these and other clinical populations, or possibly aid early diagnosis. We are currently investigating observers with Asperger’s syndrome and have preliminary results that show atypical performance in the range of spatial frequencies relevant to face processing.

One of the unique properties of CEMIT is the potential to specifically identify performance deficits at the cortical level. If participants are unable to see the stimuli clearly due to faulty optics their responding would be random (50% in the direction of the IOC) and not systematically either in the IOC direction or in the component direction. Chance performance would also be expected if the observer had an attention deficit, or when they are unable to do the task, or did not want to cooperate. Surprisingly, 90% of our observers did not fall into any of these categories. Responding correctly is also independent of retinal processing because neurones that respond to orientation do not occur at this very early stage of visual processing. CEMIT for the first time provides a way of examining cortical visual processing very precisely in a simple manner that could be carried out in a clinical environment with little training. CEMIT facilitates examination of the tuning of neurones and motion integration in early cortical visual processing. This allows research to go a step further and examine the visual cortex noninvasively. The results from CEMIT are robust and may be used to identify or characterise problems by measuring the limits of performance, or linking idiosyncratic performance with a specific clinical condition. It may also be used as a simple screening device for many researchers and clinicians who need to understand the specific contribution of fundamental cortical visual deficits in their tests or investigations.

## Appendix

The CEMIT was implemented as a comprehensive mobile app for iOS devices (iPhone, iPod touch, and iPad) with the abilities to specify and preview the stimuli properties, design and run the test, and save and export the experimental results.

The drifting plaid stimulus was generated and displayed in real time at a frame rate of 60 Hz using the OpenGL API supported by the iOS devices. The spatial properties of the stimuli were properly calibrated based on the viewing distance to the subjects and the display size and resolution for each device type. Gamma calibration is not necessary, as previous testing had shown that this does not affect the subjects’ performance; however, Gamma calibration is available. All aspects of the plaid stimulus were customised using a graphical user interface built in the mobile app: direction of the comparison, spatial frequency, aperture diameter, speed, phase jump for each plaid component, duration, and contrast. Different stimuli configurations can be set up and run in separate sessions. After each session, performance for each stimulus condition is shown in a graph, that is, as separate plotting of performance for test and control conditions as a function of the spatial frequency.

The viewing distance between the display of the iOS device to the subject was estimated using the front-facing camera of the device: The CEMIT app detects and estimates the size of the subject’s face and automatically infers the visual distance after performing a calibrating process that correlates the size of a face with the actual visual distance (roughly inversely proportional). The precision and accuracy of this estimation decreases with the visual distance, both remaining below 1 cm when holding the device at arm’s length (70 cm). The app also provides a one-point calibration process to adjust the estimation of the visual distance to account for the individual variability in face size. Moreover, to ensure that the subjects maintain a constant viewing distance, the app monitors it in real time during the session to detect and report abnormal variations in case the subject changes his/her position relative to the display. This can be used to discard data from untrustworthy sessions.

The mobile app has been designed to be as versatile as possible: Each session result can be either recorded on the device database or anonymously sent to a remote server dedicated to a large-scale analysis. In addition, the mobile app has the ability to track the subject’s face and automatically estimate the viewing distance using the front-facing camera of the device. The mobile app can also run on a secondary device then acting as a remote control (communicating through WiFi or Bluetooth) in case one needed to test viewing distances that do not allow touch-screen based inputs, or if the subject has problems providing such inputs (in this case the remote device would be operated by the experimenter to enter the subject’s responses provided orally).

The CEMIT and CEMIT Lite (this version has a fixed set of conditions) mobile apps are available on the Apple AppStore to disseminate this new test as well as to collect data from a wide range of clinical populations.
